# Multiple mediators of anxiety and depression between living space and cognitive function among elderly patients with ischemic stroke

**DOI:** 10.3389/fpsyt.2025.1682799

**Published:** 2025-11-18

**Authors:** Yu Zhang, Xinyu Yang, Qingxin Gu, Jiawen Pan, Jia Mao, Dongyan Lu, Qing Zhou, Lixiu Zhang

**Affiliations:** 1 Department of Nursing, College of Medical Science, Huzhou University, Huzhou, Zhejiang, China; 2 Huzhou Central Hospital, Affiliated Central Hospital of Huzhou University, Huzhou, Zhejiang, China; 3 Department of Nursing, Cixi People’s Hospital, Cixi, Zhejiang, China

**Keywords:** elderly, ischemic stroke, living space, anxiety, depression, cognitive function

## Abstract

**Objective:**

To explore the relationship between living space, cognitive function, anxiety, and depression in ischemic stroke patients, specifically examining the mediating roles of anxiety and depression.

**Methods:**

A cross-sectional study included 445 ischemic stroke patients treated in the neurology department of a top-tier hospital in Zhejiang Province and discharged between January 2024 and January 2025 were selected. Participants completed General information questionnaires, the Montreal Cognitive Assessment (MoCA), Living Space Scale, and Hospital Anxiety and Depression Scale (HADS). Data were analyzed using R4.4.2 and MPLUS 8.7 software.

**Results:**

Among 445 participants, correlation analysis showed that cognitive function was positively correlated with living space (*r* = 0.37, *P* < 0.05) and negatively correlated with anxiety symptoms and depression symptoms (*r* = -0.53, *r* = -0.64, all *P* < 0.05). Anxiety and depression were mediating variables between living space and cognitive function (*95%CI*: 0.018~0.117, 0.017~0.095), with effect sizes of 0.072 and 0.057, respectively. Additionally, anxiety and depression played a serial mediating role between living space and cognitive function (*95%CI*: 0.082~0.172), with an effect sizes of 0.122.

**Conclusion:**

Living space level directly affect the cognitive function in ischemic stroke patients and indirectly affects cognition through its impact on psychological health, mediated independently and serially by anxiety and depression.

## 1 Introduction

Population ageing has emerged as a leading global health challenge. Medical advances and economic development have significantly extended human life expectancy, and China is undergoing an unusually rapid demographic transition. It is predicted that by 2057, China’s elderly population will peak at 425 million, comprising 32.9%-37.6% of the total population (1). Concurrently, ischemic stroke (IS), the primary cause of death and disability worldwide, continues to impose an increasing disease burden, with an annual incidence rate of 201/100,000 in China (2). For IS survivors, long-term health management faces multiple challenges, among which post-stroke cognitive impairment (PSCI) has become an important clinical issue. PSCI affects 20%-80% of IS patients (3) and represents a prodromal stage of dementia and Alzheimer’s disease (4). Stroke related cognitive decline not only aggravates functional disability, but also elevates the risk of stroke recurrence, creating a vicious cycle of neurological deterioration. Clinically, this pathological interplay highlights a substantial individual, family, and societal burden (5).

Importantly, living space limitation, a severe limitation of mobility and community participation due to stroke-related neurological deficits, is highly prevalent in this population. Additionally, poor psychological states, particularly anxiety and depression, are common sequelae of stroke and living space limitations (6). Notably, both life-space limitations and psychological distress are potentially modifiable risk factors for cognitive decline (7). Given the lack of disease-modifying therapies for cognitive impairment, elucidation of the pathways through which these factors affect cognition, especially their potential mediating interactions, is critical for the development of targeted interventions for older stroke patients.

The life-space assessment (LSA) provides an effective framework for quantifying the spatial extent, frequency, and independence of individual movements in their environment (8, 9). Unlike traditional self-reported assessments of physical function, this model captures a spectrum of activities ranging from indoor to outdoor settings, inclusive of assistive device usage and unencumbered by physical limitations. Consequently, the Life-space model is regarded as an integrative metric for assessing mobility and social participation. Studies have indicated that lower Life-space scores are correlated with adverse outcomes such as depression, frailty, and mortality, with the degree of restriction potentially foreshadowing a decline in cognitive abilities and an increased risk of neurodegenerative diseases (6). Specifically, for post-stroke patients, life-space limitation directly impacts the scope of their daily activities and is independently associated with cognitive function decline (7). These findings highlight the association between restricted life space and cognitive decline in elderly patients post-stroke.

The limitations of living space are not just physical; It poses a significant threat to mental health. Numerous studies have shown that it is associated with increased risk of anxiety and depression in older adults and post-stroke individuals (6, 10). Anxiety and depression are potentially independent risk factors for cognitive dysfunction, impairing key brain regions such as the hippocampus and prefrontal cortex via mechanisms like neuroinflammation and oxidative stress, leading to declines in memory and executive function (11). Clinical observations indicated that post-stroke anxiety often precedes depression, with the two interacting to form an “anxiety-depression comorbidity chain.” (12) This chain may prolong the psychological recovery period for patients and, through the bidirectional reinforcement of physiological and psychological stress, increases the risk of a second stroke, creating a vicious cycle that further harms the physical and mental health of the elderly.

Despite this evidence of convergence, a significant research gap persist. Existing research frequently overlooks the distinctive characteristics of the elderly, particularly in post-stroke patients, where the influence of life-space limitations on psychological well-being and its intricate interplay with cognitive decline remain underexplored. This study aims to address this research lacuna by offering novel insights into how life-space restrictions influence cognitive function in elderly stroke patients through the lens of adverse psychological states, such as anxiety and depression.

**Figure 1:**
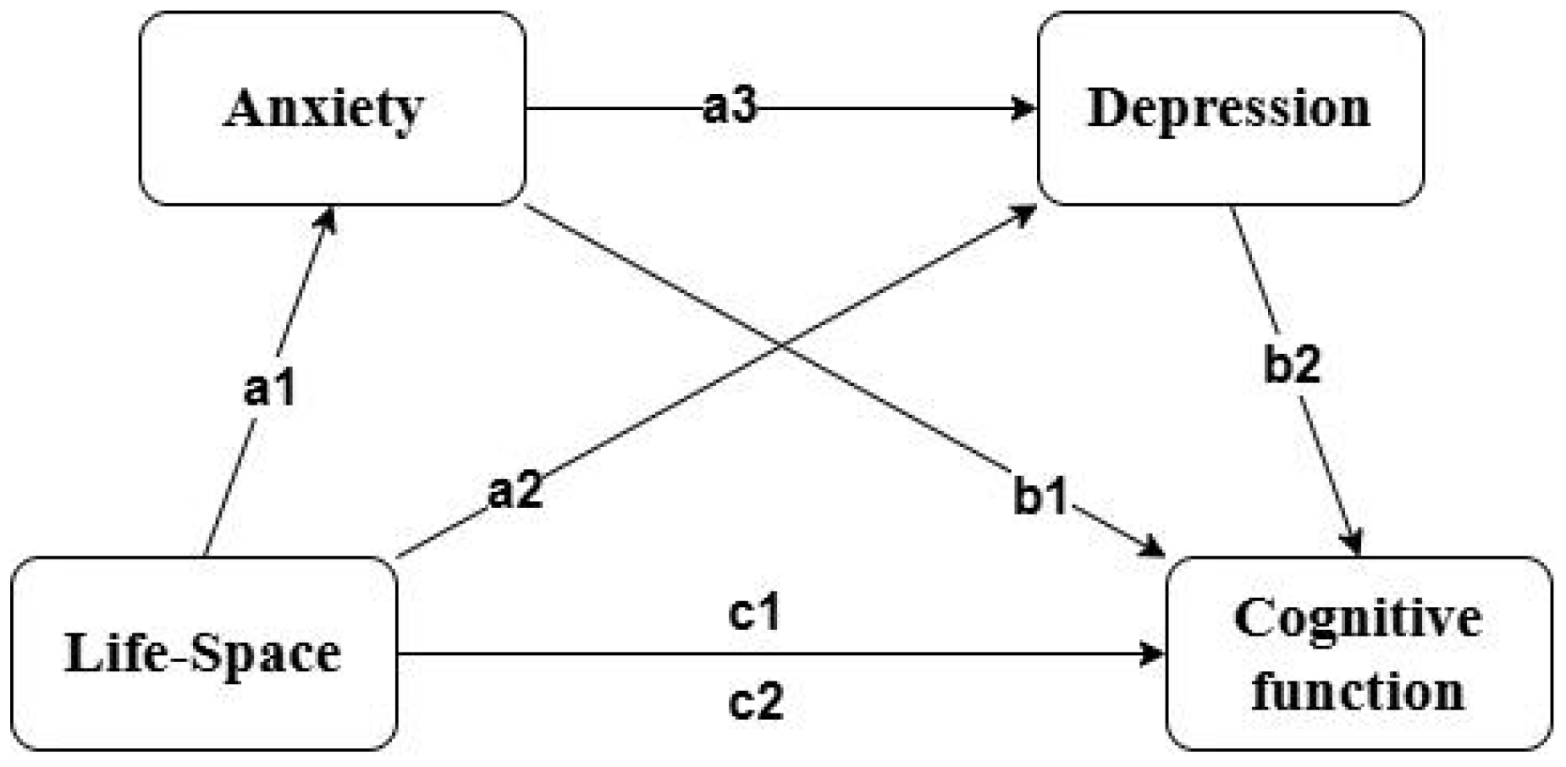
Multiple mediation model. c1 represents the total effect, while the others represent the path effects.

Consequently, the primary objective of this study is to investigate the correlation between life-space limitations and cognitive function in elderly patients with ischemic stroke while elucidating the mediating roles of adverse psychological states, including anxiety and depression. The hypotheses proposed for this study are as follows: (1) Life-space restrictions exert a direct negative influence on cognitive function; (2) Anxiety serves as a mediator in the relationship between life-space restrictions and cognitive function; (3) Depression acts as a mediator in the relationship between life-space restrictions and cognitive function; (4) Anxiety and depression may exhibit a combined mediating effect on the relationship between life-space restrictions and cognitive function (**Figure 1**).

## 2 Materials and methods

### 2.1 Data sources and subjects

This study employed convenience sampling, recruiting a specific group of elderly patients who were treated for ischemic stroke in the neurology department of a tertiary hospital in Zhejiang Province and discharged between June 2024 and December 2024. The diagnostic criteria followed were those established in the “Chinese Clinical Management Guidelines for Cerebrovascular Diseases” (13), which included patients diagnosed with ischemic stroke via cranial CT or MRI. Inclusion criteria were as follows: (1) Age≥60 years; (2) Confirmed ischemic stroke through imaging examination; (3) Ability to complete the questionnaire independently or with assistance; (4) Complete medical records; (5) Discharge time≥1 month; (6) Signed informed consent form, voluntarily participating in the study. Patients with combined malignant tumors, severe mental illness, severe functional impairment, lower limb disability, or loss of walking ability were excluded from the study.

In the context of structural equation modeling, the determination of the minimum sample size depends on the number of latent constructs within the model. Conventional guidelines suggest that a model encompassing up to five constructs necessitates a minimum sample size of 100 participants (14). Given that the present study incorporated four constructs—namely anxiety, depression, living space, and cognitive function—a total of 500 questionnaires were disseminated. Of these, 480 responses were collected, with 455 passing quality control assessments, yielding a valid response rate of 92.71%.

### 2.2 Procedure

The questionnaire survey was administered by professionals who underwent rigorous standardization training. The research team contacted patients via telephone to ascertain their willingness to return to the hospital for follow-up examinations or to receive home visits. For patients unable to physically attend, video telephony was employed for the survey. Survey locations included neurology nursing offices, outpatient clinics, patient residences, and WeChat. Ultimately, researchers conducted face-to-face interviews with participants at community healthcare centers using convenience sampling, with questionnaires distributed and completed on-site. Assistance was provided to participants requiring guidance in completing the survey.

### 2.3 Ethics considerations

Participants were fully informed about the objectives, procedures, risks, benefits, and confidentiality of the study, with the emphasis on the voluntary nature of participation. Ethical approval for the study was obtained from the Medical Ethics Committee of Huzhou University (Approval Number: 202504-19).

### 2.4 Measurements

#### 2.4.1 Cognitive function assessment

This study uses the Montreal Cognitive Assessment (MoCA) to evaluate the cognitive function of patients. The MoCA scale has a total score ranging from 0 to 30, with higher scores indicating better cognitive function (15). This scale is suitable for screening mild cognitive impairment caused by neurodegenerative diseases such as stroke and Parkinson’s disease, demonstrating high sensitivity and specificity, with a Cronbach’s α coefficient of 0.88.

#### 2.4.2 Life-space assessment scale

The Life-Space Assessment scale (LSA), translated by JI and others, was used to assess the activity range of Chinese elderly people (16). The scale evaluates spatial mobility over the past month, covering five different areas from other rooms in the home to outside the town. It integrates activity distance, frequency, and independence, with scores ranging from 0 to 120, where higher scores, indicate broader the activity space. The Cronbach’s α coefficient of the scale is 0.76.

#### 2.4.3 Hospital anxiety and depression scale

This study uses the Hospital Anxiety and Depression Scale to assess the anxiety and depression status of the subjects (17). The scale includes two subscales: the anxiety subscale and the depression subscale, with a total of 14 items scored from 0 to 3 points each. Thus, the total score of each subscale ranges from 0 to 21 points, with higher scores reflecting more severe anxiety or depression symptoms. The Cronbach’s α coefficients for the anxiety and depression subscales are 0.83 and 0.82, respectively.

#### 2.4.4 Covariates

To account for potential confounding effects, covariates included in this study were age (60–64 years, 65–69 years, 70–74 years, ≥75 years), gender (male, female), educational level (illiterate, primary school, junior high school, senior high school or vocational school, college or above), marital status (married, others), housing type (ground-floor house or single-story staircase house, elevator house with two or more floors, staircase house with two or more floors), presence of other comorbid chronic diseases (yes, no), per capita monthly household income (<3000 China Yuan (CNY), 3000–5000 CNY, >5000 CNY), primary caregiver (spouse, children, others), place of residence (urban, rural), time since stroke onset (>6 months, 3–6 months, <3 months), neurological impairment, activities of daily living (ADL), exercise self-efficacy, and family care quality.

For the assessment of neurological impairment in stroke patients, the National Institutes of Health Stroke Scale (NIHSS) was utilized. This scale produces a total score from 0 to 42, with lower scores indicating less severe neurological deficits (18). The Barthel Index was employed to gauge patients’ ADL, with a total score ranging from 0 to 100, where higher scores indicate superior self-care capabilities (19). The Chinese version of the Exercise Self-Efficacy Scale (ESES), translated and adapted by Lee (20), was used to assess exercise self-efficacy. This scale consists of nine items, each scored from 0 to 10, where 0 indicates no confidence and 10 represents the utmost confidence. The total score is calculated by summing the item scores and dividing by nine, yielding a total score range of 0 to 10, with higher scores indicating greater exercise self-efficacy. The Family Care Quality Scale(FCQS) for Stroke Patients, revised by Huang (8), was implemented in this study to assess the quality of family care. This scale encompasses 27 items across five dimensions, scored on a three-point Likert scale, with a total score range of 27 to 81, where higher scores are indicative of superior family care quality.

### 2.5 Statistical methods

Statistical analysis was conducted using R 4.4.2 software. Quantitative data were described using descriptive statistics, while count data were presented as frequencies and percentages. Comparisons between two groups were conducted using two independent sample *t*-tests, and comparisons among multiple groups were analyzed using multivariate analysis of variance. Pearson correlation analysis was used to explore relationships between variables. Harman’s single-factor test was applied to examine common method bias in the data. The significance level α was set at 0.05.

MPLUS software version 8.7 was used to construct a chained mediation model. The model included living space as the independent variable, anxiety and depression as mediator variables, and cognitive function as the dependent variable. Model fit was assessed by comparing the Comparative Fit Index(*CFI*), Tucker-Lewis Index(*TLI*), standardized root mean-square residual(*SRMR*), and root mean square error of approximation(*RMSEA*). The confidence intervals for the mediation effects were estimated using Bootstrap resampling (2000 repetitions), and if the 95% confidence interval did not include zero, the mediation effect was considered significant.

## 3 Results

### 3.1 Participant demographics

**Table 1** presents the general demographic characteristics of 445 study participants. Among them, 210 were male (47.19%) and 235 were female (52.81%). The mean age was 65.90 ± 5.835 years. Statistical analysis revealed significant differences in cognitive function among elderly patients based on age groups, educational level, housing conditions, per capita household income, primary caregivers, residential areas, and post-stroke durations (all *P* < 0.001). **Table 2** details the NIHSS scores, Barthel Index, cognitive function assessment scores, living space scores, anxiety scores, depression scores, exercise self-efficacy scores, and family care quality scores of elderly patients with ischemic stroke.

**Table 1:** Participant demographics (*n* = 445).

Variable		*n*(%)
Age	60~64 years	56(12.58)
	65~69 years	168(37.75)
	70~74 years	121(27.19)
	≥75 years	100(22.47)
Gender	Male	210(47.19)
	Female	235(52.81)
Educational attainment	Illiterate	132(29.66)
	Primary school	161(36.18)
	Junior high school	46(10.34)
	Senior high school or vocational school	62(13.93)
	College or above	44(9.89)
Marital status	Married	359(80.67)
	Others (including single, divorced, widowed, etc.)	86(19.33)
Housing type	Ground-floor house or single-story staircase house	128(28.76)
	Elevator house with two or more floors	192(43.15)
	Staircase house with two or more floors	125(28.09)
Presence of other comorbid chronic diseases	No	366(82.25)
	Yes	79(17.75)
Per capita monthly household income	<3000 CNY	161(36.18)
	3000~5000 CNY	225(50.56)
	>5000 CNY	59(13.26)
Primary caregiver	Spouse	212(47.64)
	Children	179(40.22)
	Others(relatives, caregivers, etc.)	54(12.13)
Place of residence	Urban	317(71.24)
	Rural	128(28.76)
Time since stroke onset	>6 months	104(23.40)
	3~6 months	254(57.1)
	<3 months	87(19.60)

**Table 2:** Scores of various scales (*n* = 445).

Variable	Score range	Score ( $\bar{x}\pm s$)
NIHSS	0-42	12.89 ± 6.52
Barthel	0-100	58.51 ± 25.88
Cognitive function	0-30	20.41 ± 5.95
Life-Space	0-120	55.23 ± 11.12
Anxiety	0-21	7.33 ± 4.35
Depression	0-21	6.89 ± 4.02
Exercise self-efficacy	0-10	4.86 ± 1.39
Family care quality	21-81	67.97 ± 23.69

### 3.2 Correlation analysis

**Figure 2:**
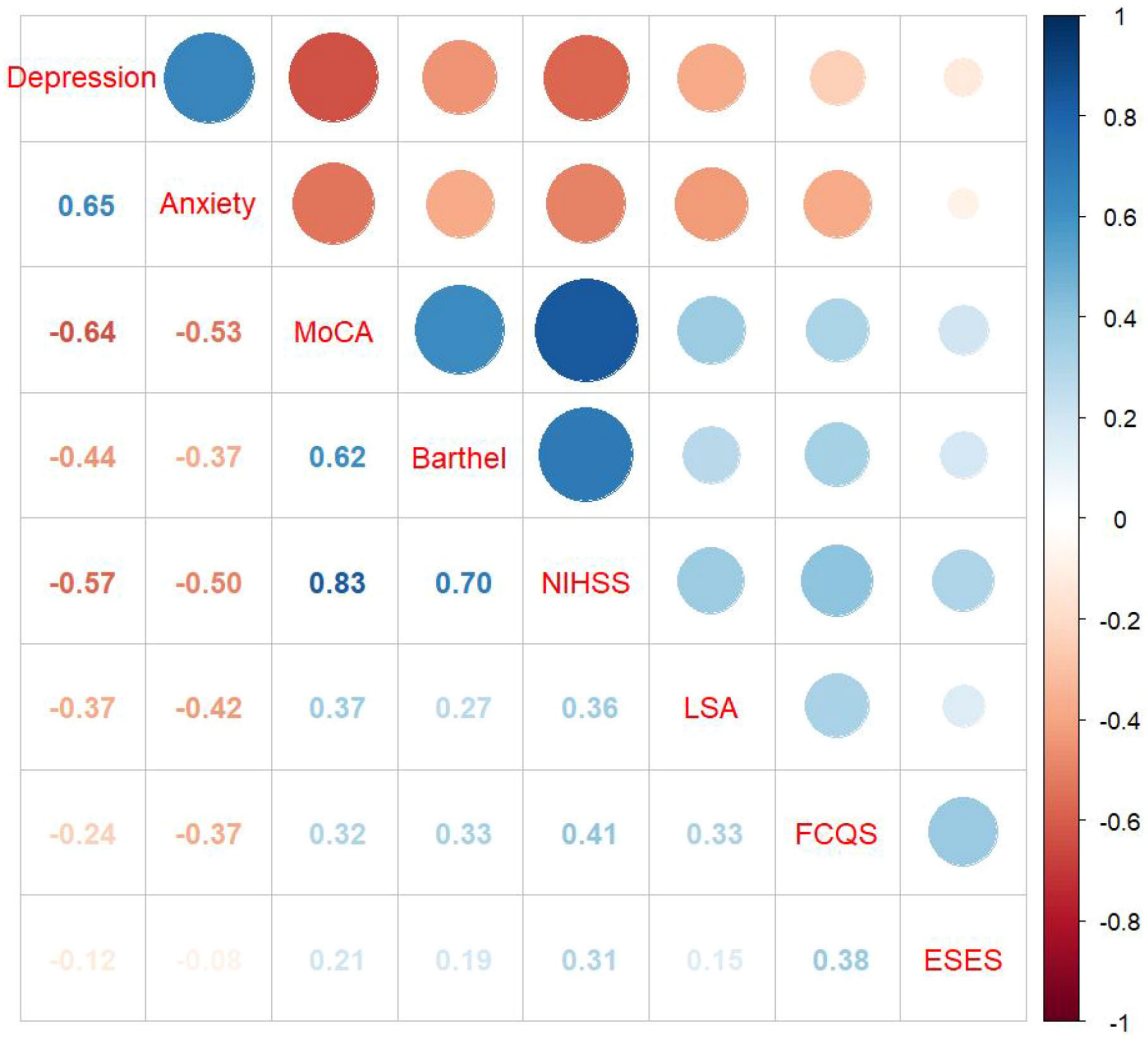
Correlation heatmap. Specific values indicate *P* < 0.05; MoCA: Montreal Cognitive Assessment; ESES: Exercise Self-Efficacy Scale; FCQS: Family Care Quality Scale; LSA: Life-Space Assessment scale.

A correlation analysis was conducted on the cognitive function, living space, anxiety, and depression of elderly ischemic stroke patients. Results indicated that depression and anxiety scores were negatively correlated with cognitive function scores (*r* = -0.64, *P* < 0.001; *r* = -0.53, *P* < 0.05) and living space scores (*r* = -0.37, *P* < 0.001; *r* = -0.42, *P* < 0.05). Conversely, the living space score was positively correlated with cognitive function score (*r* = 0.37, *P* < 0.001). For specific results, see **Figure 2**.

### 3.3 Multiple mediation effects

This study employed structural equation modeling (SEM) to investigate the mediating effects of anxiety and depression on the relationship between living space and cognitive function in elderly individuals post-stroke. After controlling for potential confounding factors such as age, educational attainment, housing conditions, household per capita monthly income, primary caregiver, residential area, post-stroke duration, NIHSS index, Barthel index, motor self-efficacy, and family care quality, the model demonstrated a good fit(*CFI* = 1, *TLI* = 1, *SRMR<*0.001, *RMSEA<*0.001). To verify the significance of the mediating effects, a non-parametric percentile Bootstrap method was utilized, with 2000 resamples and a 95% confidence interval (*CI*) calculated. Path coefficients are detailed in **Table 3**. Results showed that the total effect of living space on cognitive function was 0.366 (*P* < 0.05), with a total mediating effect of 0.251 (*P* < 0.05). After controlling for mediating variables, the direct effect of living space on cognitive function was 0.115 (*P* < 0.05). Anxiety and depression played a partial mediating role between living space and cognitive function, with mediating effects of 0.072 (*P* < 0.05) and 0.057 (*P* < 0.05), respectively, and a combined mediating effect of 0.122. Effect size comparisons revealed that Path 3 > Path 1 > Path 2 (all *P* < 0.05), indicating that the serial mediating effect of anxiety and depression was greater than their independent mediating effects, with the independent mediating effect of depression being the smallest.

**Table 3:** Mediation analysis (*n* = 445).

Path		*β(95%CI)*	*SE*	*P*
Total effect	c1	0.366(0.241~0.477)	0.061	<0.001
Direct effect	c2	0.115(0.007~0.221)	0.053	<0.001
	a1	-0.421(-0.496~ -0.343)	0.008	<0.001
	a2	-0.118(-0.193~-0.040)	0.008	<0.001
	a3	0.601(0.519~0.675)	0.008	<0.001
	b1	-0.170(-0.283~-0.043)	0.006	<0.001
	b2	-0.483(-0.612~-0.375)	0.008	<0.001
Indirect effect		0.251(0.198~0.298)	0.025	<0.001
Path1	X→M1→Y	0.072(0.018~0.117)	0.003	0.004
Path2	X→M2→Y	0.057(0.017~0.095)	0.001	0.004
Path3	X→M1→M2→Y	0.122(0.082~0.172)	0.001	0.032

X, Life-Space; M1, Anxiety; M2, Depression; Y, Cognitive function.

## 4 Discussion

This study investigates the interrelationships and mechanisms of interaction between living space, symptoms of anxiety and depression, and cognitive function. The results indicate that, among the elderly population post-stroke, symptoms of anxiety and depression mediate the relationship between living space and cognitive function, with their indirect effects accounting for 19.6% and 15.57% of the total effects, respectively. Furthermore, symptoms of anxiety and depression exhibit multiple mediating effects between living space and cognitive function in the elderly post-stroke, with these effects constituting 33.33% of the total effects. This suggests that living space limitation in elderly patients with ischemic stroke may lead to cognitive impairment by exacerbating mental health issues such as anxiety and depression.The study’s cross-sectional design means that the observed relationships are merely associative and do not allow for the inference of causality. Further validation of the causal pathways should be sought through longitudinal or experimental research in the future.

Compared with the study of Sartori et al. in the general elderly population (21), this study found that the influence of living space on cognitive function of elderly patients after stroke is more significant (21). This enhanced impact may be attributed to limited mobility and rehabilitation needs of elderly people after stroke. Expanding living space requires higher cognitive participation, while environmental stimulation can promote neural plasticity. In contrast, living space expansion in the general elderly population often relies on daily routines, which offer lower cognitive stimulation intensity. Studies suggest that a higher level of living space can provide elderly individuals with more abundant opportunities for social interaction and cognitive stimulation, which are not only crucial for maintaining and enhancing cognitive function but also provide essential emotional support, reduce loneliness, and stimulate brain vitality (8). As living space increases, patients have more opportunities to engage in physical activities, which can promote cerebral blood circulation, enhance neural plasticity, and have a positive effect on cognitive function (22, 23). Furthermore, optimizing living space can help improve patients’ psychological states, enabling them to cope with stress more effectively and maintain the stability of cognitive function (3). It is important to note that there may be a bidirectional relationship between cognitive function and living space limitations, where a decline in cognitive function may lead to impaired orientation, restricting living space, and in turn, potentially reducing cognitive stimulation (24). Therefore, the causal relationship between them still needs to be further verified by prospective studies.

Studies have shown that the positive effect of living space expansion on cognitive function of the elderly after stroke may be achieved by increasing environmental stimulation and improving social participation, while anxiety and depression symptoms may weaken or amplify this process. In terms of individual mediating effects, anxiety and depression independently impact the relationship between living space and cognitive function through distinct mechanisms. Anxiety diminishes the willingness of patients to explore their living space, for instance, by reducing outings due to fear of falling or social embarrassment, leading to a decrease in environmental stimulation and, consequently, a decline in cognitive function (25). On the other hand, depressive symptoms reduce motivation and energy levels, diminishing the enthusiasm of patients to engage in complex environmental interactions, thereby limiting living space, reducing cognitive stimulation, and leading to decreased neural plasticity (10, 26).

This study reveals for the first time the multiple mediation effects among living space constraints, anxiety, depression, and cognitive function, identifying a cascade pathway “living space reduction → anxiety → depression → cognitive decline”. Specifically, the constriction of living space may induce anxiety, and sustained anxiety, through the hyperactivation of the hypothalamic-pituitary-adrenal (HPA) axis (27), triggers neuroinflammation, which in turn leads to depression and ultimately affects cognitive function (28). Neuroimaging research supports this pathway, showing that anxiety-related amygdala hyperactivity is associated with abnormalities in the default mode network related to depression, both contributing to hippocampal volume reduction and frontal lobe functional inhibition, thereby accelerating cognitive impairment (29, 30). Although the path coefficients (*β*) in this study are statistical measures, their magnitude and significance provide crucial insights into the interplay between various factors and their potential clinical implications. In the model of this study, the total effect of living space on cognitive function is 0.366, indicating that expanding the living space is a clinically valuable intervention target for improving cognitive function in elderly stroke patients. Specifically, regarding the mediating pathways, the independent mediating effects of anxiety and depression (*β* = 0.072 and *β* = 0.057), while not large in absolute terms, both achieve statistical significance. This suggests that even if there are limited objective improvements in living space, targeted alleviation of patients’ anxiety or depressive symptoms can still have an independent and positive effect on their cognitive function. The discovery of this mediation mechanism has significant clinical implications, suggesting that alleviating anxiety through cognitive-behavioral therapy or improving mood with antidepressant medications. For the cascade mediation pathway, early identification and interruption of barriers by improving the living environment and combining mindfulness training to regulate emotional responses may be the key intervention strategies to improve cognitive decline in the elderly after stroke.

In summary, the mediating pathways quantified in this study provide a theoretical model and data support for the development of multi-layered, precision rehabilitation intervention strategies. Future intervention studies can estimate sample sizes based on the effect sizes of this study and aim to verify the effectiveness of the following integrated approach: on one hand, encouraging and assisting patients to safely expand their living space (such as participating in community activities and taking outdoor walks) to gain direct cognitive stimulation; on the other hand, providing concurrent psychological health support, especially early intervention for anxiety, to maximize the mitigation of cognitive risks associated with limited living space. By adopting this dual-pronged “environment-psychology” approach, it is expected to more effectively maintain the cognitive health of elderly patients with ischemic stroke and enhance their long-term quality of life.

Through structural equation model and Bootstrap mediation analysis, this study constructs a multiple mediation model of “space-psychology-cognition” in stroke population for the first time, which makes up for the limitations of traditional single-factor research. By employing a multi-dimensional analysis, the study not only uncovers the impact of environmental factors on the cognitive function of elderly patients with ischemic stroke but also delves into how psychological states modulate this influence. This provides a scientific basis for the development of targeted environmental improvements and psychological interventions.

While this study focuses on the elderly population following ischemic stroke, several limitations must be acknowledged, particularly given the rapid growth of this vulnerable demographic. Firstly, the cross-sectional design precludes the elucidation of causal relationships, suggesting that future longitudinal follow-up or intervention studies are needed to verify the causal role of mediating pathways. Secondly, reliance on self-reported questionnaires may introduce recall bias. For the sake of research feasibility and clinical applicability, future studies should incorporate objective indicators (such as neuroimaging, blood biomarkers, smart wearable devices, etc.) to enhance the objectivity and accuracy of the data. Lastly, the study sample is limited to participants from Zhejiang Province, which inherently restricts the generalizability of the results to other regions in China. Future research should conduct multicenter, multiregional, and cross-cultural studies to increase the universality and applicability of the findings.

## 5 Conclusion

In summary, elderly patients who have suffered from ischemic stroke can engage in self-regulation by altering their living space levels, which may mitigate anxiety and depressive states, thereby influencing cognitive function. This study underscores the importance for the elderly to expand their living spaces and actively participate in a variety of enriching activities. Maintaining good mental health by alleviating symptoms of anxiety or depression can be beneficial in preventing or decelerating the decline in cognitive function and enhancing the quality of life. In the future, multi-center prospective studies combined with objective measurement methods are needed to further verify the causal relationship, so as to provide a more solid evidence basis for the development of interdisciplinary intervention strategies.

## Data Availability

The original contributions presented in the study are included in the article/supplementary material. Further inquiries can be directed to the corresponding authors.
